# Inhibition of HMG CoA reductase reveals an unexpected role for cholesterol during PGC migration in the mouse

**DOI:** 10.1186/1471-213X-8-120

**Published:** 2008-12-31

**Authors:** Jiaxi Ding, DeChen Jiang, Michael Kurczy, Jennifer Nalepka, Brian Dudley, Erin I Merkel, Forbes D Porter, Andrew G Ewing, Nicholas Winograd, James Burgess, Kathleen Molyneaux

**Affiliations:** 1 Department of Genetics Case Western Reserve University, Cleveland, OH, USA; 2 Department of Chemistry, Case Western Reserve University, Cleveland, OH, USA; 3 Department of Chemistry, Penn State University, University Park, PA, USA; 4 Program on Developmental Endocrinology and Genetics, Eunice Kennedy Shriver National Institute of Child Health and Human Development, National Institutes of Health, Bethesda, MD, USA; 5 Department of Chemistry, Gothenburg University, Kemivägen 4, SE-41296 Gothenburg, Sweden

## Abstract

**Background:**

Primordial germ cells (PGCs) are the embryonic precursors of the sperm and eggs. Environmental or genetic defects that alter PGC development can impair fertility or cause formation of germ cell tumors.

**Results:**

We demonstrate a novel role for cholesterol during germ cell migration in mice. Cholesterol was measured in living tissue dissected from mouse embryos and was found to accumulate within the developing gonads as germ cells migrate to colonize these structures. Cholesterol synthesis was blocked in culture by inhibiting the activity of HMG CoA reductase (HMGCR) resulting in germ cell survival and migration defects. These defects were rescued by co-addition of isoprenoids and cholesterol, but neither compound alone was sufficient. In contrast, loss of the last or penultimate enzyme in cholesterol biosynthesis did not alter PGC numbers or position in vivo. However embryos that lack these enzymes do not exhibit cholesterol defects at the stage at which PGCs are migrating. This demonstrates that during gestation, the cholesterol required for PGC migration can be supplied maternally.

**Conclusion:**

In the mouse, cholesterol is required for PGC survival and motility. It may act cell-autonomously by regulating clustering of growth factor receptors within PGCs or non cell-autonomously by controlling release of growth factors required for PGC guidance and survival.

## Background

Primordial germ cells (PGCs) are the embryonic precursors of gametes. In most model systems, PGCs are migratory and navigate through or around diverse tissues in order to find the site of the developing gonads. PGC migration shares conserved features in many species indicating the process arose in a common ancestor. In vertebrates, however, the majority of factors implicated in PGC guidance are either secreted or membrane bound protein growth factors (e.g. stromal derived factor 1 and Kit ligand); whereas, evidence in Drosophila points to a lipid-based guidance system [1]. A recent study bridged the gap by demonstrating that zebrafish PGCs, like Drosophila PGCs, require 3-hydroxy-3-methylglutaryl-coenzyme A reductase (HMGCR) for normal migration [2].

HMGCR is the rate limiting enzyme in isoprenoid and cholesterol biosynthesis. In flies, HMGCR was shown to act within the somatic gonadal precursors to control release of a secreted PGC attractant [3]. Drosophila lack enzymes required downstream of HMGCR for cholesterol synthesis indicating that isoprenoids are the relevant downstream effectors [4]. In support of this, Santos and Lehmann demonstrated that mutations in the geranylgeranyl transferase 1 β subunit cause PGC migration defects. It has been proposed that geranylgeranylation of small GTPase in the Ras, Rac, or Rho families regulate secretion of hedgehog [5] or other putative Drosophila PGC attractants [4]. In zebrafish, evidence also points to a role for HMGCR and isoprenoids in PGC migration [2]. Inhibition of HMGCR or geranylgeranyl transferase I cause PGC migration defects. However, in this system it remains unclear whether HMGCR is required in PGCs themselves or within the soma. Additionally, zebrafish unlike flies are capable of de novo cholesterol synthesis and this branch of the pathway was not carefully evaluated.

Cholesterol plays a vital role during vertebrate development. Mutations in genes required for cholesterol biosynthesis cause severe developmental defects. Loss of *Hmgcr *[6] or *squalene synthase *[7] result in early embryonic lethality in mouse models. Mutations in *3b-hydroxysterol-Δ7 reductase *(*Dhcr7*) or *lathosterol 5-desaturase *(*Sc5d*) cause skeletal, neural, and in some cases urogenital defects in humans [8] and in mice [9,10]. Additionally, mutations in genes required for cholesterol transport are also associated with embryonic lethality or patterning defects [11].

Several models have been evoked in order to explain the role of cholesterol during organogenesis. First, cholesterol is the precursor of steroid hormones, glucocorticoids, and oxysterols, all compounds known to mediate cell-cell signaling via activation of nuclear hormone receptors. Second, cholesterol directly regulates cell-cell signalling by controlling the diffusion [12] or reception [13] of members of the hedgehog growth factor family. Finally, cholesterol is a key structural component of the plasma membrane. Cholesterol controls membrane fluidity and modulates membrane protein interactions. Membrane cholesterol has been shown to influence the activity of growth factor receptors and cell-adhesion molecules by clustering these cell surface proteins into lipid rafts [14]. Of particular note, lipid rafts have been shown to affect epidermal growth factor-induced chemotaxis [15] and migration on fibronectin [16] in cell culture. This suggests that cholesterol levels might also alter cell migration in vivo.

Considering the roles of cholesterol in cell-cell signalling and cell migration, we thought it imperative to test whether this branch of the HMGCR pathway is required for germ cell development in a vertebrate model. In mice, PGCs migrate from the gut to the genital ridges between embryonic day 9.5 (E9.5) and E10.5. Steroid hormones and hedgehog growth factors are unlikely to play a role in this process. Enzymes required to convert cholesterol into steroid hormones are not expressed in the gonads until E11.5 [17]. Likewise, the three vertebrate members of the hedgehog growth factor family are not expressed in the right place or time to play a role in PGC guidance in this system [18,19]. However, changes in cholesterol could very well impact the ability of PGCs to respond to proposed chemoattractants such as SDF1 [20] or KITL [21].

**Figure 1:**
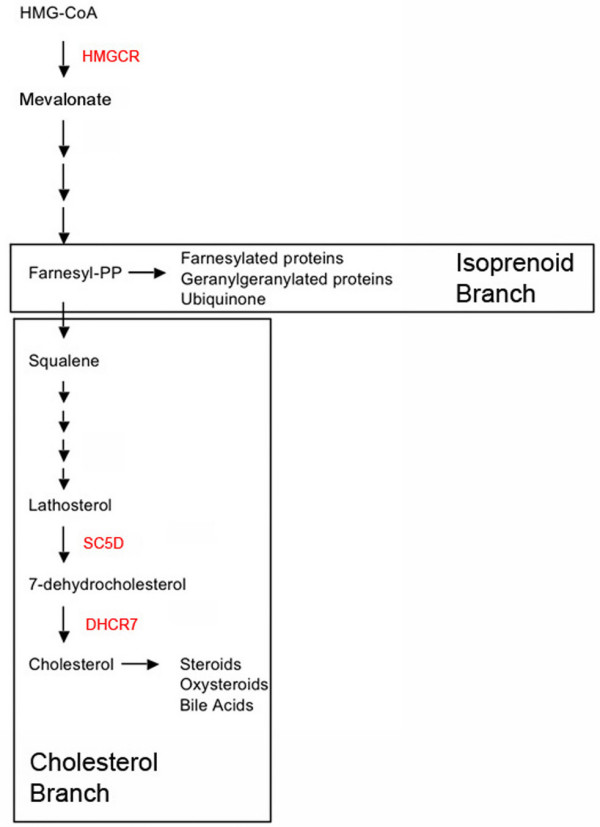
**The HMGCR pathway**. HMGCR is the rate-limiting enzyme that converts HMG-CoA into mevalonate. Mevalonate serves as a precursor for the synthesis of both cholesterol and isoprenoids. Enzymes inhibited in this study are indicated in red.

To examine the role of cholesterol in PGC migration, we first measured cholesterol in living tissue dissected from E9.5 embryos and were surprised to find that cholesterol was enriched in the genital ridges relative to the surrounding tissue. This asymmetric distribution appears to be maintained via selective uptake of cholesterol by cells within the genital ridges. We further demonstrate that inhibition of HMGCR reduced total cholesterol and impaired germ cell survival and migration in culture. These defects were rescued by co-addition of geranylgeraniol and cholesterol indicating that both compounds are required. To test if cholesterol biosynthesis is necessary for PGC migration in vivo, we examine the number and distribution of PGCs in embryos lacking *Dhcr7 *or *Sc5d *(see Figure 1). PGCs were normally distributed in both lines, but cholesterol levels are only modestly affected in these embryos [9,10]. We conclude that cholesterol is required for PGC migration but that this requirement can be met by uptake of cholesterol from maternal sources. We propose that the asymmetric accumulation of cholesterol within the genital ridges controls signaling interactions required for PGCs to colonize the gonads.

## Results

### Cholesterol is elevated in the genital ridges

**Figure 2:**
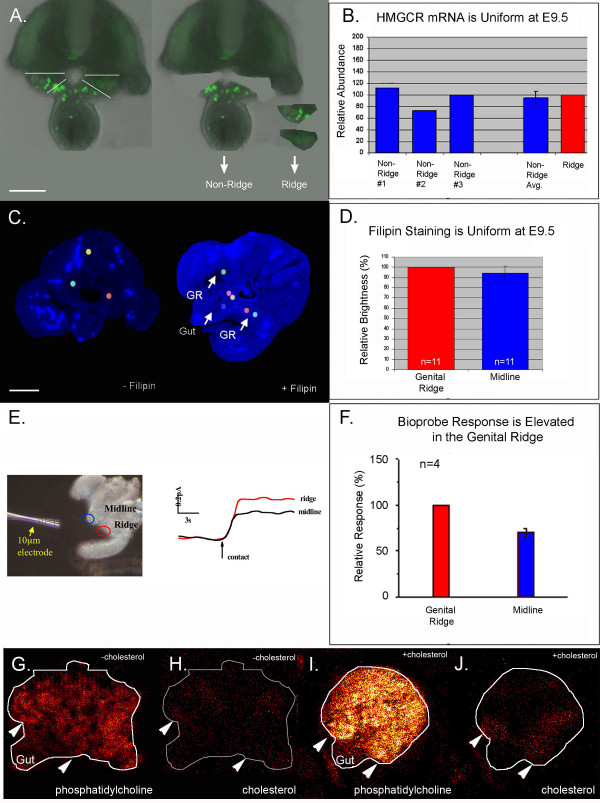
**Cholesterol uptake is elevated in the genitalridges**. (A) A transverse tissue slice dissected from an E9.5 embryo. Genital ridge and non-ridge tissue were isolated as shown. (B) *Hmgcr *expression in ridge and non-ridge tissue was similar in three independent quantitative RT-PCR experiments. Ridge *Hmgcr *levels were set to an arbitrary value of 100. (C) Unstained (background fluorescence) and Filipin stained E9.5 tissue. (D) The average filipin staining intensity of midline and ridge tissue was calculated by measuring pixel intensity in three midline and three lateral (colored spots in C) regions per slice and normalizing to the average ridge staining (set to 100%). (E) A cholesterol bioprobe gave an elevated response when touched to ridge as compared to midline tissue. (F) Summary of bioprobe data taken from four slices. Midline response was normalized to the ridge response (100%). Scanning TOF-SIMS of an E9.5 slice imaged for (G) phosphatidylcholine (m/z = 184) or (H) cholesterol (m/z = 369). E9.5 tissue pre-treated for 30 minutes with soluble cholesterol and imaged for (I) phosphatidylcholine or (J) cholesterol. Arrowheads indicate the position of the genital ridges. In all panels "n" = number of slices and error bars are s.e.m. Scale bars are 100 μm.

*Drosophila Hmgcr *mRNA is elevated in somatic gonadal precursors [3] supporting the model that HMGCR acts non-cell autonomously to guide PGC migration. However, zebrafish *Hmgcr2 *is uniformly expressed in cleavage stage and gastrulating embryos, raising the possibility that HMGCR has both non-autonomous and autonomous effects [2]. Like in zebrafish, mouse *Hmgcr *is ubiquitously expressed in E10.5 embryos based on *in situ *hybridization [22]. However, *in situs *are not sensitive enough to reveal modest changes in gene expression. As a more sensitive technique, quantitative RT-PCR was used to compare *Hmgcr *mRNA levels within the genital ridge and non-ridge tissue during PGC migration (E9.5) (Figure 2A and 2B). Mouse *Hmgcr *was uniformly expressed at this stage confirming the previous *in situ *hybridization results.

HMGCR activity is controlled by transcriptional and post-transcriptional mechanisms. For example, cholesterol and its oxysterol derivatives feedback and inhibit *Hmgcr *expression at the transcriptional level [23], while both steroids and isoprenoids inhibit HMGCR activity by inducing degradation of the HMGCR protein [24]. Therefore, the absolute amount of *Hmgcr *mRNA is unlikely to be an accurate indicator of the activity of the pathway. The distribution of cholesterol was examined to provide an indirect measure of HMGCR pathway activity (Figure 2C–F). In initial experiments, filipin staining [25] was used to map the distribution of cholesterol during PGC migration (Figure 2C and 2D). Filipin is a naturally fluorescent antibiotic that binds unesterified cholesterol. Filipin staining was uniform at E9.5; however we were concerned that fixation and processing (e.g. permeabilization) might have caused diffusion of cholesterol in the samples. To avoid processing artifacts, we took advantage of both an electrochemical method developed to measure plasma membrane cholesterol levels in living cells [26-28] as well as time of flight secondary ion mass spectrometry (TOF-SIMS) [29] (Figure 2E–J). A cholesterol oxidase tipped bioprobe was used to compare surface cholesterol levels within the genital ridge and midline tissues (Figure 2E and 2F). Current generated by the probe is proportional to the cholesterol level at the contact site of the probe which has a tip diameter of 10 μm. The bioprobe detected a moderate, but consistent elevation of cholesterol within the genital ridge relative to the midline (gut mesentery). TOF-SIMS analysis performed on tissue slices that were snap frozen immediately after dissection did not detect a consistent elevation of cholesterol in the ridges (Figure 2G, H). However, accumulation of cholesterol in the genital ridges was detected when the embryonic tissue slice was incubated for 30 minutes in soluble cholesterol prior to freezing (Figure 2I and 2J). This suggests that cells within the genital ridges accumulate high levels of cholesterol via uptake instead of de novo synthesis. We propose that localized uptake of cholesterol within the ridge modulates the signalling interactions required for early development of the gonad.

### Inhibition of HMGCR causes germ cell and somatic cell apoptosis

**Figure 3:**
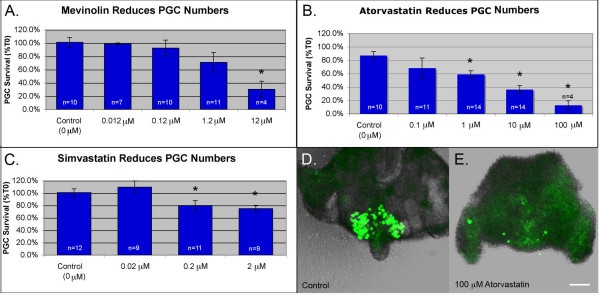
**Statin treatment reduces PGC survival**. PGCs were counted in tissue cultured in (A) mevinolin (B) atorvastatin or (C) simvastatin. Average percent survival ± s.e.m. is shown. n = number of slices. "* " = groups that differed from controls. (F < 0.05 Analysis of Variance from Walpole and Meyer with Fishers least significance post test). (D) A control slice after culture. (E) A slice treated with 100 μM atorvastatin. Note sloughing of somatic cells from the surface of the slice. Scale bar is 100 μm.

Cells in the developing embryo can obtain cholesterol via either de novo synthesis or via uptake. This makes it challenging to manipulate cholesterol levels in utero. To test the role of cholesterol in PGC migration, we have used an organ culture system in which PGC containing tissue is allowed to develop in cholesterol-free media. This eliminates the possibility of uptake from maternal sources and allows us to manipulate cholesterol levels simply by inhibiting synthesis. To test the role of cholesterol in PGC development, tissue slices dissected from E9.5 embryos were cultured in the presence of statins, drugs that act as competitive inhibitors of the HMGCR enzyme. Dose response curves were established for mevinolin, simvastatin and atorvastatin (Figure 3A–C and Additional file 1). All three statins caused a dose dependent decrease in PGC numbers after 18 hours. 0.2 μM simvastatin and 1 μM atorvastatin reduced PGC survival to 80% and 59% respectively but mevinolin was only effective at comparatively high doses. 12 μM mevinolin was the lowest dose of this drug found to induce a statistically significant drop in PGC survival. High doses of atorvastatin were toxic. 100 μM atorvastatin reduced PGC survival to 13% (Figure 3C) and caused sloughing of the somatic cells from the surface of the tissue slices (Figure 3D and 3E).

**Figure 4:**
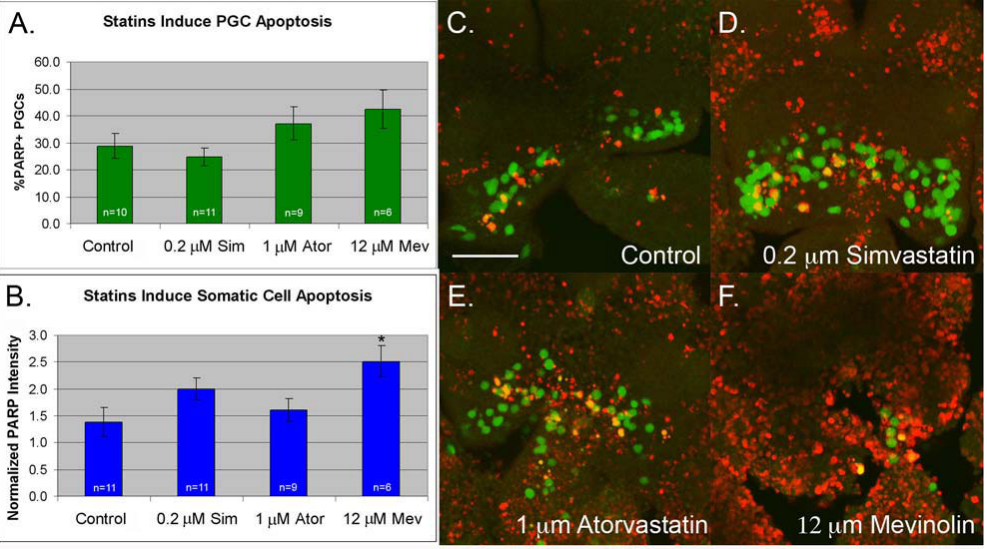
**Statin treatment induces both somatic cell and germ cell apoptosis**. E9.5 tissue was treated with statins, fixed and stained for apoptotic cells (cleaved PARP+). (A) The % of apoptotic PGCs (PARP+. GFP+) per slice was calculated. (B) The amount of somatic cell apoptosis was compared by summing the pixel intensity of the PARP staining and normalizing to an arbitrarily chosen control slice (value set to 1). (C) Apoptosis in a control slice (D) a slice treated with 0.2 μM simvastatin (E) a slice treated with 1 μM atorvastatin and (F) a slice treated with 12 μM mevinolin. In all panels "n" = number of slices, error bars are s.e.m. and " * " = groups that differed from controls (F < 0.05 Analysis of Variance from Walpole and Meyer with Fishers least significance post test). Scale bar is 100 μm.

To examine whether statin-treatment affects total cell survival, we compared the level of apoptosis in statin treated and untreated slices (Figure 4). Both atorvastatin and mevinolin induced a slight increase in PGC apoptosis. This trend was not statistically significant and may reflect the fact that immunostaining was performed at the end of the assay (T18), a timepoint at which many of the PGCs had already died and been cleared. In support of this, time lapse analysis of atorvastatin treated slices revealed that germ cells fragment and disappear at an average rate of 0.8 PGCs per hour (Additional files 2, 3 and 4). PGC apoptosis was not elevated in the simvastatin-treated samples consistent with the weaker effect of this drug on PGC survival (see Figure 3). However, both simvastatin and mevinolin caused an increase in somatic cell apoptosis. Mevinolin-treatment was particularly toxic, causing a two fold increase in cleaved PARP signal. Of the three statins, atorvastatin had the most potent and specific effect on PGCs. The 1 μM dose reduced PGC numbers without causing an increase in somatic cell apoptosis.

**Figure 5:**
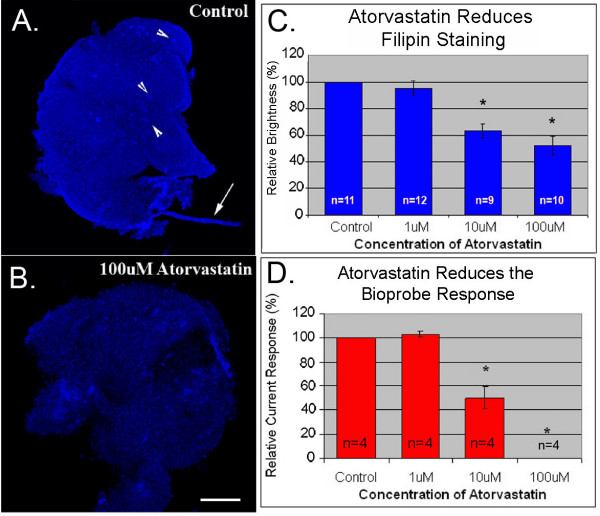
**Statin treatment reduces total cholesterol levels**. (A) Filipin staining of control tissue. Arrow heads indicate autofluorescent cells and arrow indicates lint. (B) Filipin staining of tissue treated with 100 μM atorvastatin. (C) Average filipin staining intensity in treated and control slices normalized to control values (set to 100%). (D) Average bioprobe response in treated and control tissue normalized to controls (set to 100%). Error bars are s.e.m.. n = number of slices. " * " = groups that differed from controls (F < 0.05 Analysis of Variance from Walpole and Meyer with Fishers least significance post test). Scale bar is 100 μm.

Recent evidence suggests that statins can elicit responses that are independent of their effects on HMGCR [30]. Additionally, the effects of inhibiting HMGCR itself may be complex, involving modulation of isoprenoids, cholesterol or both. To test whether our treatment was effective at inhibiting HMGCR, we used filipin staining and the cholesterol oxidase tipped bioprobe to compare cholesterol levels in atorvastatin-treated and untreated tissue. Atorvastatin-treatment caused a dose dependent reduction in both filipin staining and current response (Figure 5). 10 μM atorvastain was the lowest dose capable of causing a statistically significant drop in cholesterol levels. It is interesting to note that 1 μM atorvastatin had little effect on cholesterol levels despite causing a significant reduction in germ cell numbers. This indicates that germ cells may be more sensitive to inhibition of HMGCR than the surrounding tissue.

### PGC migration requires both cholesterol and isoprenoids

**Figure 6:**
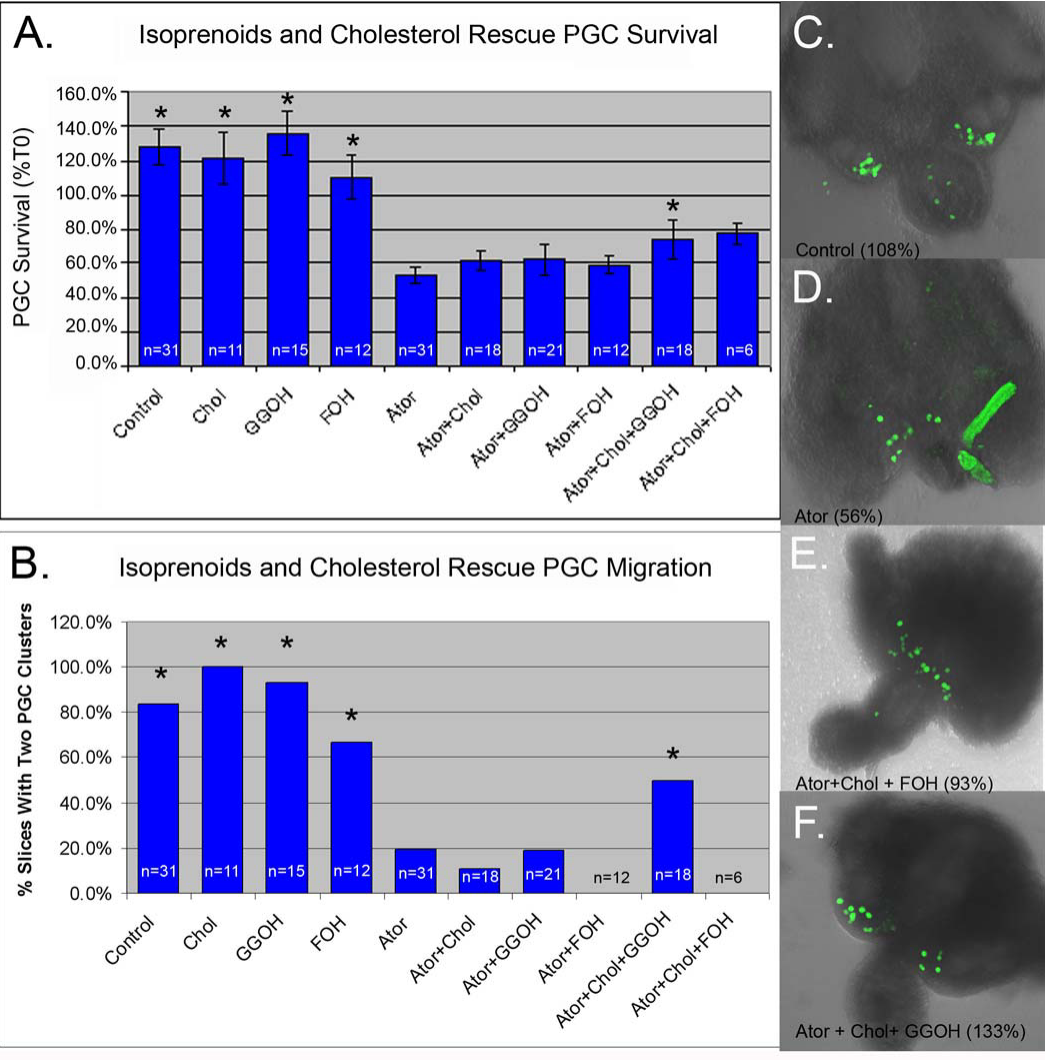
**Cholesterol and isoprenoids rescue statin-induced PGC survival and migration defects**. (A) Average PGC survival in tissue treated with the indicated combinations of cholesterol (1× concentration), GGOH (pooled data from 10 uM and 20 uM treatments), FOH (10 uM) and atorvastatin (10 uM). " * " = groups that differed from the atorvastatin group (F < 0.05 Analysis of Variance from Walpole and Meyer with Fishers least significance post test). (B) The percentage of slices in each treatment group that had two PGC clusters after culture. "*" = groups that differed significantly from the atorvastatin group (exact binomial test P-value < 0.05) (C) Control slice that exhibited robust migration (two distinct PGC clusters) and survival (108%). (D) Atorvastatin treated slice with scattered PGCs and reduced survival (56%). (E) Slice treated with cholesterol, FOH and atorvastatin with excellent survival (100%), but poor migration. (F) Slice treated with atorvastatin, cholesterol and GGOH with robust migration and survival (133%). Scale bar is 100 μm. Error bars are s.e.m. n = number of slices.

Rescue experiments were used to test if isoprenoids and cholesterol have independent roles during PGC migration (Figure 6). In these rescue experiments, atorvastatin was used at 10 μM, the lowest dose found to reduce cholesterol levels in organ culture (Figure 5). Tissue dissected from E9.5 embryos was treated with atorvastatin alone, with atorvastatin and isoprenoids (geranylgeraniol or farnesol), with atorvastatin and cholesterol, or with atorvastatin, isoprenoids and cholesterol. Cholesterol, geranylgeraniol or farnesol alone had a very modest and not statistically significant protective affect (Figure 6A). However, co-treatment of slices with cholesterol and geranylgeraniol or with cholesterol and farnesol partially rescued PGC survival (Figure 6A).

While performing these experiments, it was noted that statin-treated slices often had PGCs accumulating on the midline whereas PGCs in control slices normally cleared the midline and formed two distinct clusters at the genital ridges (Figure 6C). Treated slices were filmed and this effect was found to be caused by a reduction in PGC velocity (Additional files 2, 3 and 4). Neither cholesterol nor isoprenoids alone were sufficient to rescue this defect in PGC motility but co-treatment of slices with both geranylgeraniol and cholesterol was able to rescue this defect (Figure 6B, E and 6F). Co-treatment with farnesol and cholesterol did not rescue PGC migration. In fact farnesol treatment in any combination appeared to inhibit normal migration (Figure 6B).

### De novo cholesterol synthesis is not required for PGC migration in vivo

Our in vitro culture data suggests that both isoprenoids and cholesterol are necessary for PGC survival and motility. To test the role of cholesterol in germ cell development in vivo, we examined the distribution of PGCs in embryos lacking DHCR7 or lacking SC5D, the last two enzymes in the cholesterol biosynthetic pathway (Figure 1). These animals exhibit late gestational deficits in cholesterol levels, but are still able to obtain some cholesterol via uptake from maternal sources. We could not examine PGCs in animals lacking HMGCR because a loss of this enzyme results in early embryonic lethality [6]. Animals lacking DHCR7 die shortly after birth and exhibit reduced body weight, reduced motility, and a failure to feed [10]. Animals lacking SC5D, the second to last enzyme in cholesterol synthesis are more severely effected. They are stillborn and exhibit skeletal malformations [9].

**Figure 7:**
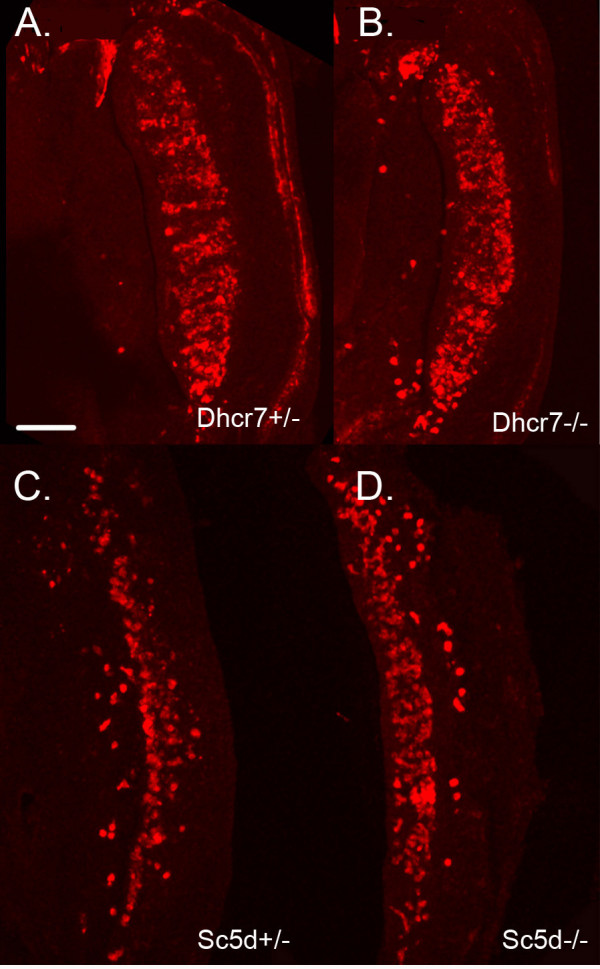
**Loss of de novo cholesterol synthesis does not perturb PGC migration**. E12.5 gonads from (A) a *Dhcr7*+/- embryo and (B) a *Dhcr7*-/- littermate. Gonads were stained for the PGC marker SSEA1. Similar results were seen for four *Dhcr7-/- *embryos derived from two litters. E12.5 gonads dissected from (C) an *Sc5d *+/- embryo and (D) an *Sc5d*-/- littermate. Similar results were seen for seven *Sc5d-/- *embryos derived from four litters. Scale bar is 146 μm.

Gonads were dissected from E12.5 *Dhcr7*-/-, *Sc5d*-/- embryos and their littermate controls and the numbers of PGCs compared by immunostaining for SSEA1. There were no obvious defects in PGC number or position in these animals (Figure 7). This does not rule out a possible role for cholesterol in PGC guidance. *Dhcr7*-/- mice were shown to have normal liver cholesterol levels at E12.5 and only exhibit cholesterol deficiency at later stages [10]. Also, *Sc5d-/- *mice, despite having a more severe spectrum of malformations than the *Dhcr7-/- *mice, exhibit similar late gestational reductions in total cholesterol [9]. This demonstrates that the cholesterol requirement for early post-implantation development can be provided via the yolk sac or placenta. Perturbing cholesterol levels during PGC migration in vivo will require interventions affecting both synthesis and uptake of cholesterol.

## Discussion

This study demonstrates that cholesterol is required for primordial germ cell survival and motility. Inhibition of HMGCR reduced cholesterol levels and induced PGC apoptosis in culture. Addition of cholesterol and farnesol or cholesterol and geranylgeraniol rescued germ cell survival; however, PGC motility was only rescued by the latter combination. Additionally, we found that cholesterol is elevated in the urogenital ridges and present evidence that this asymmetric distribution can be maintained by differential uptake. In support of this, embryos lacking the last or penultimate enzyme in cholesterol biosynthesis do not have germ cell defects but these embryos do not exhibit cholesterol deficits until late in development [9,10]. We conclude that the cholesterol requirement for early developmental processes including PGC migration can be met by uptake of maternal cholesterol.

HMGCR and isoprenoids are required for migration of cardiac progenitors and PGCs in both fly and zebrafish model systems [2,4,31,32]. In these systems, isoprenylation of heterotrimeric G-protein subunits and/or isoprenylaton of small G-proteins in the Ras superfamily are thought to be altered by loss of HMGCR activity resulting in the observed developmental phenotype. Our rescue experiments demonstrate that isoprenoids may play a similar role during PGC migration in mammals and demonstrate differential roles for GGOH and FOH. Both farnesol and geranylgeraniol co-treatments were able to rescue PGC survival, but only geranylgeraniol co-treatment assisted migration. This probably reflects differential isoprenylation requirements for different small GTPase [33]. For instance, Ras proteins are typically farnesylated, but when farnesylation is inhibited some Ras family members can be geranylgeranylated. Likewise, the small GTPase RhoB can be modified by either isoprenyl group, but the selection of group has a profound effect on its subcellular localization and presumably function. We propose that either farnesylation or geranylgeranylation can support signalling via a Ras family member involved in controlling PGC survival or proliferation. However, geranylgeranyl modification is required to support the activity of a small GTPase (perhaps in the Rho family) required for cell motility.

In addition to reflining what has already been shown about the function of HMGCR and isoprenoids in PGC development, our data also hints at a function for cholesterol in PGC survival or motility. A role for cholesterol during gonadal development is not entirely without precedent. First, genes known to coordinate cholesterol uptake are elevated within the urogenital ridges (UGRs). Steroidogenic factor 1 (*Nr5a1*) is expressed in the UGRs at E9.5 and its expression becomes confined to the testis by E12.5. NR5A1 is a member of the nuclear receptor family and controls expression of genes required for cholesterol synthesis (*HmgCoA synthase*) and uptake (*Scarb1*) as well as genes required for steroid production [34]. *Scarb1 *mRNA has been detected in the sexually naive genital ridge as early as E10.5 and like *Nr5a1*, it later become enriched in the testis [17]. Loss of *Nr5a1 *results in loss of *Scarb1 *expression in the UGRs [35] and an absence of gonads and adrenal glands in both male and female mice [36]. Second, in the adult ovary, genes required for cholesterol synthesis are elevated within the granulosa cells surrounding the oocyte and cholesterol synthesized by the soma helps support oocyte growth by metabolic coupling [37]. Curiously, migratory PGCs appear to lack mevalonate kinase and mevalonate decarboxylase enzymes required for isoprenoid and cholesterol biosynthesis [38]. This suggests that migratory germ cells are already deficient in cholesterol synthesis and may rely on interactions with the soma to supply their metabolic needs.

**Figure 8:**
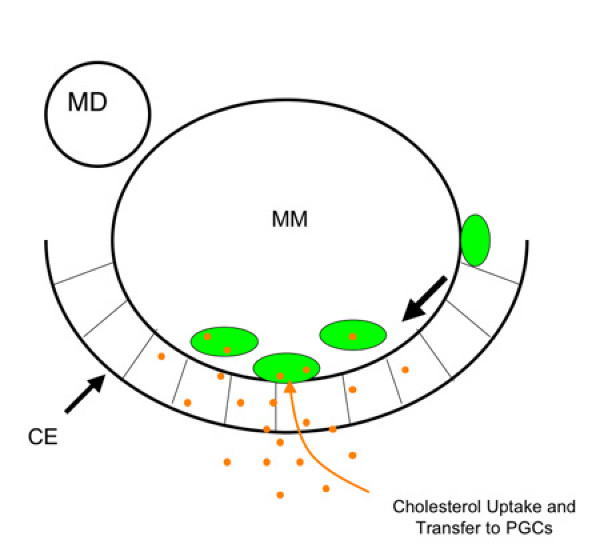
**A non-cell autonomous model for the role of cholesterol in PGC development**. Cholesterol (orange) taken up or synthesized by cells of the coelomic epithelium (CE) is transferred to PGCs (green). This helps offset the absence of metabolic enzymes in PGCs and it facilitates the formation of lipid rafts thus amplifying the ability of PGCs to respond to locally produced growth factors or chemokines. MM indicates mesonephric mesenchyme and MD the mesonephric duct. Large arrow indicates the direction of migration.

Cholesterol alters development via its ability to regulate cell-cell signaling. Cholesterol is covalently attached to members of the hedgehog (HH) growth factor family and this modification controls diffusion of the HH proteins [12]. Additionally, cholesterol is required for cells to respond to HH and evidence suggests that it is this process that is perturbed by mutations in *Dhcr 7 *[13]. In flies, HH is a proposed attractant for PGCs [39] but in mice, the expression pattern of hedgehog family members [18,19] does not suggest a role in PGC guidance. Additionally, a screen for transcripts expressed in migratory PGCs failed to detect expression of *Gli *genes [38] transcription factors required for HH response. We prefer a model in which changes in cholesterol alter the secretion or reception of growth factors known to be required for mammalian PGC migration. For instance cholesterol rich lipid rafts have been shown to be required for reception of KITL [40] and SDF1 [41] growth factors implicated in PGC guidance [20,21]. Alternatively, the cholesterol rich environment within the genital ridge might help support PGC survival via metabolic coupling [37] (Figure 8).

## Conclusion

In summary, HMGCR and its downstream products isoprenoids and cholesterol are required for mammalian PGC survival and motility in organ culture. However, in vivo support for this awaits the development of a system for efficiently manipulating cholesterol levels in utero. The role of HMGCR and isoprenoids in PGC migration has been well established in fly and zebrafish systems but this is the first study reporting a role for cholesterol in this process. Additionally, we have demonstrated that cholesterol preferentially accumulates in the genital ridges. This observation suggests that cholesterol may play a non-cell autonomous role in PGC development by either controlling secretion of growth factors required for PGC migration or by regulating development of the somatic support cells of the gonads. This study provides insight into how changes in cholesterol (through diet or genetics) might contribute to changes in development that ultimately impact fertility later in life.

## Methods

### Organ culture experiments

All animal procedures were approved by the Case Western Institutional Animal Care and use Committee. Embryos heterozygous for the Oct4:ΔPE:GFP germ cell marker were generated by crossing Oct4:ΔPE:GFP [42] males with CD1 females (Charles River). Embryonic day 0.5 (E0.5) was assumed to be noon on the day on which a cervical plug was seen. On E9.5, pregnant females were sedated with isoflurane and sacrificed by cervical dislocation. The uterus was removed and placed into phosphate buffered saline (PBS). Embryos were dissected from the uterus using forceps and then transferred via pipette into DMEM/F-12 media (Invitrogen) supplemented with 100 U of penicillin,100 mg streptomycin (Invitrogen) and 0.04%lipid free BSA (Sigma Chem. Co.) (culture media). Transverse slices approximately 2 somites thick were cut from the trunk region using a scalpel. Dissected tissue was placed into organ culture chambers (MiliCel) pre-coated with collagen IV (Beckton-Dickinson). The culture chambers were incubated overnight at 37°C in 24-well plates containing 700 ml per well culture media with or without additives. Atorvastatin (Toronto Research Chemicals), Simvastatin (EMD Chemicals) and Mevinolin (Sigma Chem. Co.) stock solutions were prepared in methanol. Geranylgeraniol (GGOH) (Sigma Chem. Co.) and farnesol (Sigma Chem. Co.) stocks were prepared in 2:1 chloroform:methanol. Stock solutions were diluted into culture media in order to give 10 μM FOH, 10 μM GGOH or 20 uM GGOH. The 10 uM and 20 uM doses of GGOH were not found to be statistically different in rescue experiments hence this data was pooled for final analysis. Where appropriate, a similar amount of carrier (methanol or chloroform:methanol) was added to the media of the control samples. Soluble cholesterol (SyntheChol™) (Sigma Chem. Co.) was purchased and used at 1× working concentration as per the manufacturer's instructions.

Germ cell numbers were quantified using a Leica TCS SP2 AOBS filter-free Confocal Laser Scanning microscope. Each slice was optically sectioned (at 5 μm intervals) after 0 hours (T0) and eighteen hours (T18) in culture. Germ cell numbers were counted in the T0 and T18 pictures using Velocity Image analysis software (Improvision). The % change in germ cell number for each slice equals T18/T10*100.

### Time lapse analysis

Tissue was cultured at 37°C on the stage of the Leica TCS SP2 AOBS Confocal system. A single optical section was captured every 9 minutes for 15 hrs. (100 frames). The automated tracking feature of Velocity Image analysis software was used to track PGC movements. Tracking parameters were as follows. First, PGCs were identified by percent intensity within the GFP channel (Lower:40 Upper:100). Next, holes were filled in the identified objects. Touching objects were separated based on size (using a 100 μm^2 ^size guide). Then any small particles (< 40 μm^2^) were excluded and large PGC clumps that had failed to be separated were also excluded (> 300 μm^2^). Tracking was performed on the identified objects using the shortest path algorithm with a maximum of a 10 μm distance between nodes. Velocity is typically only able to follow a given cell for part of a movie before it looses track of the object due to changes in brightness, clumping, moving out of focus, death etc. To accommodate this, the resulting traces were sorted based on the length of time that Velocity was able to follow the individual particles. Velocity measurements from the 20 temporally longest traces were averaged to give an average cell velocity for each film. Average trace times were 3.2 ± 0.17 s.e.m. hours for control slices (n = 80 traces in four films) and 2.9 ± 0.11 s.e.m. for atorvastatin treated slices (n = 170 traces in seven films).

### Cholesterol measurements

Mouse tissue was fixed in 4% paraformaldehyde in PBS rocking overnight at 4°C. The tissue was then washed 10 minutes at room temperature in 1.5 mg/mL glycine (in phosphate buffered saline with 0.1% triton × 100 (PBST)) to block any active aldehyde groups which might otherwise contribute to autoflourescence. The tissue was rinsed three times in PBST and rocked overnight at 4°C with PBST to permeabilize the tissue. Slices were incubated with 0.05 mg/mL filipin (Sigma Chem. Co.) at room temperature for 30 minutes rocking and were then washed three times with PBST (15 minute per wash), incubated in 1:1 glycerol:PBS (10 minutes) and mounted in. 4:1 glycerol:PBS (10 minutes). Filipin staining was visualized by confocal microscopy using the UV laser at 360–370 nm excitation and 425 nm emission. Filipin staining intensity was quantified for each slice using Veloocity imaging software (Improvision). The background signal (the average signal of the -filipin slices) was subtracted and the raw signal normalized to control values (set to 100%).

For bioprobe measurements, platinum microelectrodes were fabricated in house (11.5 μm and 100 μm diameter wire, Goodfellow Corp.) as described [26]. Platinum wire was inserted into glass capillaries (Kimax-51, Kimble products) and placed inside a heated platinum coil. The glass was pulled to create a thin insulating layer on the platinum wire. The capillary microelectrodes were polished using a beveling machine (WPI, Inc.) to produce a disk electrode. The microelectrodes were immediately immersed in a 5 mM hexane solution of 11-mercaptoundecanoic acid (95%, Aldrich Chem. Co) for 2 hours to form a carboxylic acid terminated monolayer on the electrode surface. Then, the microelectrodes were treated with 2 mM 1-ethyl-3-(3-dimethylaminopropyl) carbodiimide (Sigma Chem. Co.) in 100 mM PBS solution (pH 7.4) for 30 minutes to activate the carboxyl groups to an acylisourea intermediate. The modified electrode was immersed in 1 mg/ml recombinant cholesterol oxidase (Oriental Yeast Co. Ltd., 42.0 units/mg) solution for 3 hours allowing this intermediate to react with amine immobilizing the enzyme on the electrode surface.

Amperometric measurements were conducted using a two-electrode cell and a voltammeter-amperometer (Chem-Clamp, Dagan corp.). The three-pole bessel filter in voltammeter-amperometer was set to 100 Hz. The output was further processed using a noise-rejecting voltmeter (model 7310 DSP, Signal Recovery Inc.) to digitally filter 60-Hz noise. An Ag/AgCl (1 molar KCl) reference electrode was used for all experiments, and the applied potential is 780 mV versus the normal hydrogen electrode for all experiments. All experiments were performed in 100 mM phosphate buffer (pH 7.4) at 36°C. Excised tissue was captured by a capillary prepared in house using an IM-6 microinjector (Narishige International USA, Inc.). The electrode was initially positioned about 50 μm from the tissue for acquisition of baseline data. The electrode was repositioned for contacting the biological sample and acquisition of electrode response.

Secondary ion mass spectrometry images were acquired using a time of flight secondary ion mass spectrometer (TOF-SIMS) described elsewhere [43]. The instrument utilizes 40 keV C_60_^+ ^ion source (Ionoptika, LTD.). The pulsed primary ion source was operated at an anode voltage of 40 kV angled at 40° to the sample. The beam was focused to approximately 1 μm in diameter, and delivered 10 pA of current in 50 ns pulses. Images were acquired by rastering the pulsed primary ion beam across the sample region and collecting a mass spectrum for each pixel in the image. Using imaging software written in-house, molecule-specific images were created by selecting a mass peak of interest from the summed total mass spectra and plotting the intensity of this mass at each pixel in the image.

SIMS imaging was carried out under ultra high vacuum requiring that the samples be free of volatile water before analysis. For this consideration samples were freeze-dried. Briefly the tissue slices they were rinsed in distilled water, frozen in liquid N_2 _and fixed to a copper sample stub, which was also cooled to liquid N_2 _temperature. The stub and the sample were entered into vacuum chamber (10^-8 ^torr) and allowed to warm for several hours until all the water had sublimed.

### Whole-mount immunostaining

E9.5 slices or E12.5 whole embryos were fixed in 8% paraformaldehyde in PBS rocking overnight at 4°C. The tissue was rinsed three times in PBST and the embryonic gonads were removed from the E12.5 samples. Tissue slices or whole gonads were permeabilized overnight at 4°C in PBST. Tissue was then blocked overnight at 4°C in 2% goat serum/2% Ig-G free BSA (Jackson Immuno Research) in PBST. Samples were then incubated at 4°C overnight in primary antibodies (1:100 anti-cleaved PARP) (Cell Signaling Technology), or 1:500 SSEA1 (Developmental Studies Hybridoma Bank)) diluted in block followed by five 1 hour room temperature washes with PBST. Samples were incubated at 4°C overnight in 1:200 dilution of secondary antibodies (goat anti-rabbit IgG Cy5 or goat anti-mouse IgM AlexaFluor 647) (Jackson Immuno Research) in block followed by five 1 hour room temperature washes with PBST. The tissue was then cleared by incubation in 1:1 glycerol:PBS (10 minutes) followed by 4:1 glycerol:PBS (10 minutes) and mounted in Vectashield mounting media. Samples were left overnight at 4°C to allow the Vectashield to completely penetrate and then the staining was visualized using confocal microscopy. For long-term storage of some whole embryo samples, the tissue was dehydrated through a methanol series and stored at -20°C prior to the antibody staining procedure described above. Methanol treatment did not adversely affect either cleaved PARP staining or SSEA1 staining.

### RT-PCR for HMGCR mRNA

Quantitative RT-PCR was performed as described [44]. Briefly, total mRNA was isolated from mouse tissue using TRIzol (Invitrogen) and linear polyacrylamide (Sigma Chem Co.) as a carrier. cDNA was prepared using the Superscript III kit (Invitrogen). Primers against *Hmgcr *(F: CACCTCTCCGTGGG TTAAAA and R: GAAGAAGTAGGCCCCCAATC), Tata-binding protein (F: CTTCGTGCAAGAAATGCTGA and R:AGAACTTAGCTGGGAAGCCC) and β-Actin (F: AGAGGGAAATCGTGCGTGAC and R: CAATAGTGATGACCTGGCCGT) were designed using Primer 3 . The genital ridge cDNA was left undiluted or diluted 1:2 and 1:10 to generate a 3 point standard curve corresponding to 100, 50 and 10 arbitrary expression units. The relative expression level of *Hmgcr *and loading controls (Tata-binding protein and β-actin) in the non genital ridge tissue was compared to the genital ridge standard curve using real time PCR. QuantiTect SYBR Green Mix (Qiagen) was used as a source of Taq, Buffer and dNTPs. The Chromo4 system (MJ Research) was used to perform the cycling, fluorescent measurements and melting curves. Cycling conditions were 1) 95°C for 15 minutes, 2) 40× (denature (95°C 30 sec), anneal (51°C 30 sec.), extend (72°C 30 seconds), plate read (74°C for 30 seconds followed by plate read)) 3) incubate at 72°C for 5 minutes, 4) melting curve from 70°C to 95°C, read every 1°C. Raw expression units for *Hmgcr *were normalized using the average of the loading controls (tata-binding protein and β-actin).

## Abbreviations

BSA: bovine serum albumin; DHCR7: 3b-hydroxysterol-Δ7 reductase; E9.5: embryonic day 9.5; GFP: green fluorescent protein; HH: hedgehog; HMGCR: 3-hydroxy-3-methylglutaryl-coenzyme A reductase; KITL: kit ligand; NR5A1: steroidogenic factor 1; PARP: poly(ADP-ribose) polymerase 1; PBS:phosphate buffered saline; PGC: primordial germ cell; RT-PCR: real time polymerase chain reaction; SC5D: lathosterol 5-desaturase; SCARB1: scavenger receptor class b member 1; SDF1: stromal derived factor 1; TOF-SIMS: time of flight secondary ion mass spectrometry; UGR:urogenital ridge.

## Authors' contributions

JD performed and quantified the statin treatments, filipin staining and PARP staining. The initial draft of the results section was prepared as part of her undergraduate honors thesis. DJ performed the bioprobe measurements. The current response data was analyzed by DJ and JB. MK, AE and NW performed TOF-SIMS analysis. JN checked plugs and maintained the Oct4ΔPE:GFP mouse colony. BD prepared the genital ridge and midline cDNA samples for real time PCR. EM and FP isolated and genotyped the *Dhcr7 *and *Sc5d *embryos originally generated by FP. KM performed the SSEA1 staining, rescue experiments and time lapse experiments and drafted the manuscript.

## Supplementary Material

Additional file 1 **Statin treatment reduces PGC survival.** Average PGC numbers ± s.e.m in statin-treated and control tissue at the start and end of culture. This data is presented in Figure 3 as the % PGC survival. Click here for file

Additional file 2 **Time lapse analysis of PGC behaviour in atorvastatin treated tissue.** Slices were cultured with (7 slices) or without (4 slices)10 uM atorvastatin and filmed for 15 hrs. (A) In atorvastatin-treated slices, PGCs were observed to fragment (arrow) and disappear. (B) The kinetics of PGC loss were determined by counting cells every 1.5 hrs. (C) Atorvastatin also slowed PGC migration. "n" = number of cells that were tracked. Error bars are s.e.m. "**" indicates a sample that differed from controls (Student's t-test p < 0.005). Click here for file

Additional file 3 **PGC migration in control tissue.** Time lapse of PGC behaviour in control tissue cultured for fifteen hours. Click here for file

Additional file 4 **PGC migration in atorvastatin treated tissue.** Time lapse of PGC behaviour in tissue treated with 10 μM atorvastatin and cultured for fifteen hours. Click here for file
