# Supplementary figures and images for: Inhibition of HMG CoA reductase reveals an unexpected role for cholesterol during PGC migration in the mouse
Source: BMC Dev Biol. 2008 Dec 31;8:120. doi: 10.1186/1471-213X-8-120 (PMC2631600; doi:10.1186/1471-213X-8-120)

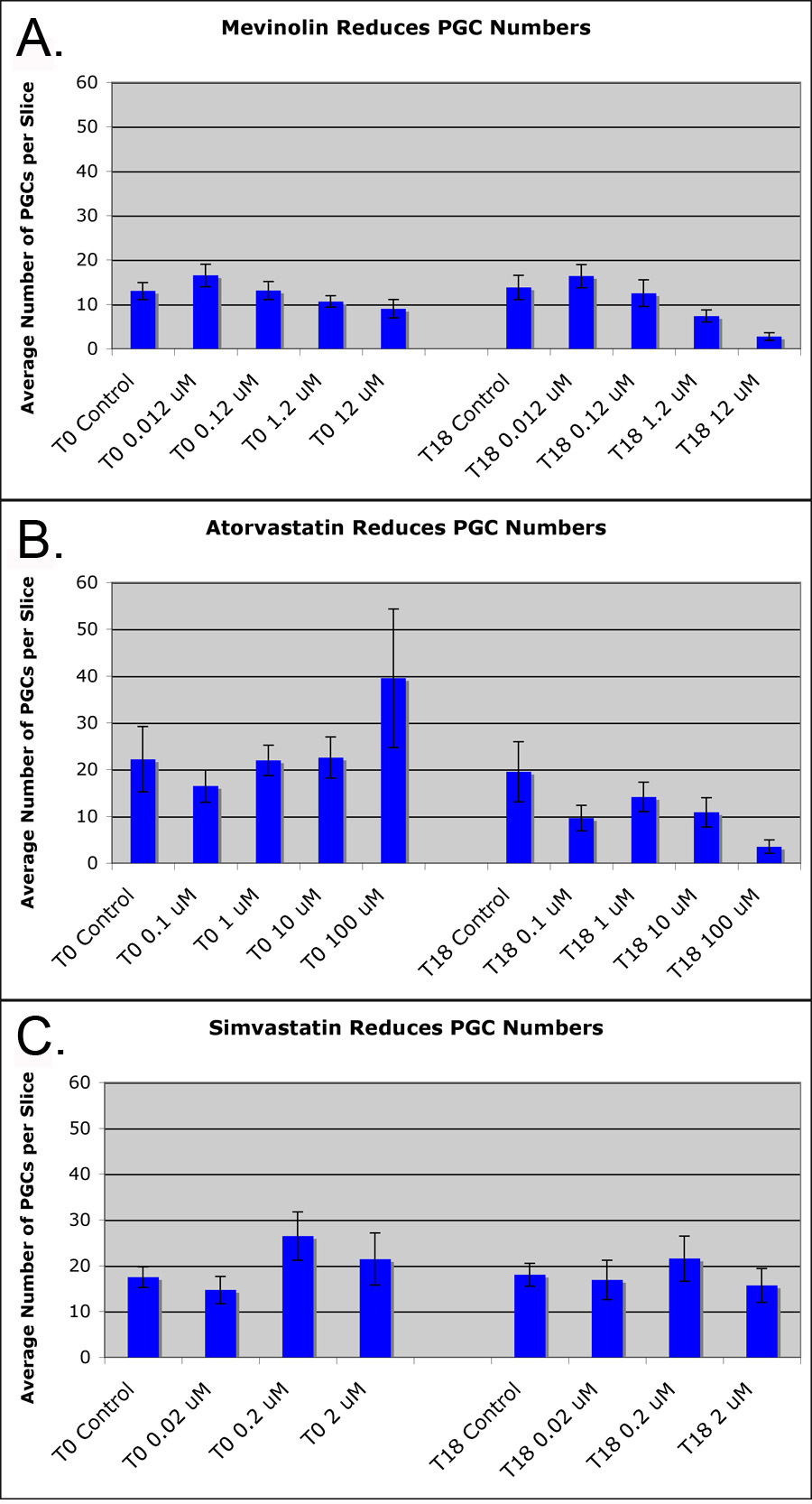

Supplement: Additional file 1 — Statin treatment reduces PGC survival. Average PGC numbers ± s.e.m in statin-treated and control tissue at the start and end of culture. This data is presented in Figure 3 as the % PGC survival. [file 1471-213X-8-120-S1.jpeg]

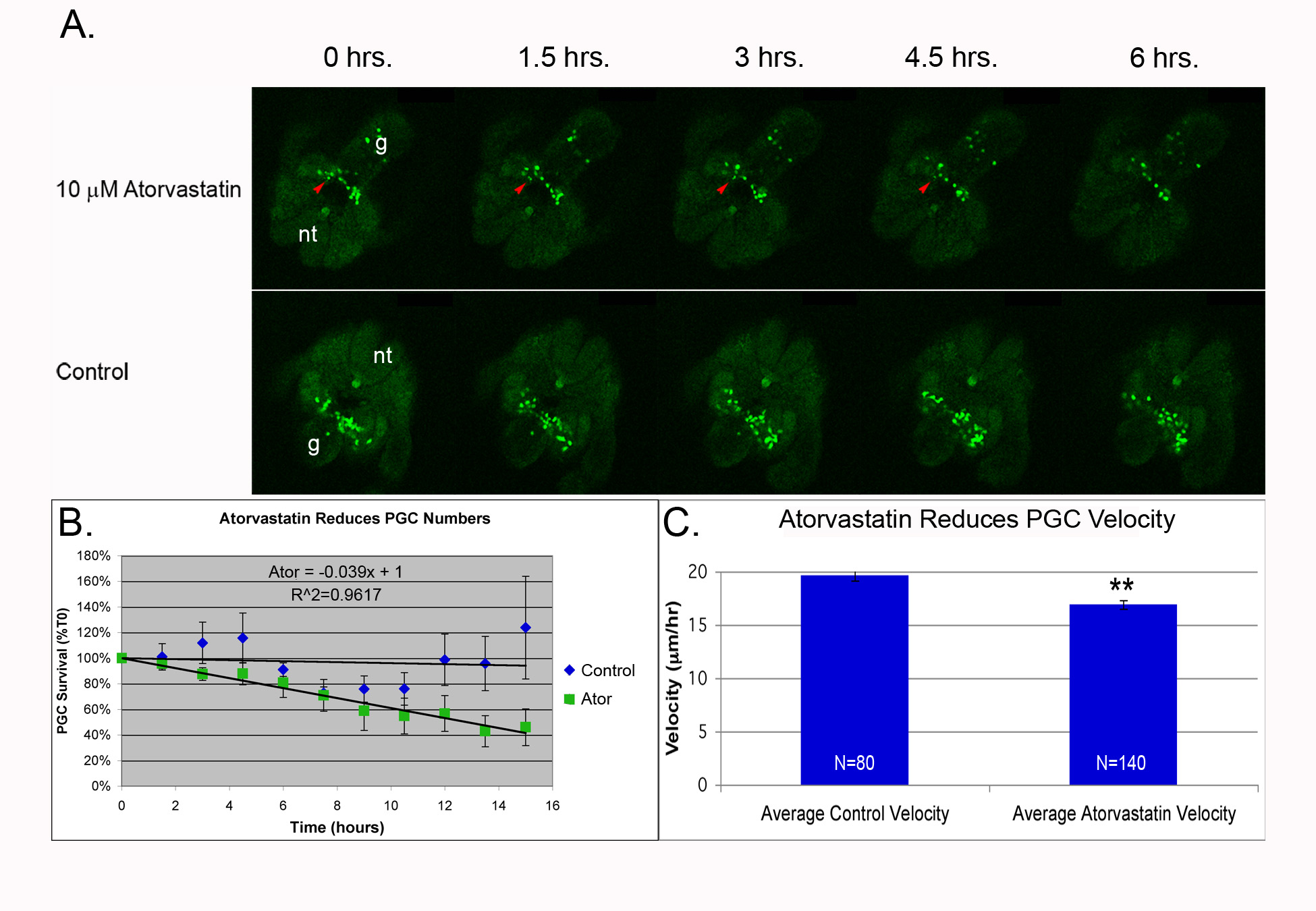

Supplement: Additional file 2 — Time lapse analysis of PGC behaviour in atorvastatin treated tissue. Slices were cultured with (7 slices) or without (4 slices)10 uM atorvastatin and filmed for 15 hrs. (A) In atorvastatin-treated slices, PGCs were observed to fragment (arrow) and disappear. (B) The kinetics of PGC loss were determined by counting cells every 1.5 hrs. (C) Atorvastatin also slowed PGC migration. "n" = number of cells that were tracked. Error bars are s.e.m. "**" indicates a sample that differed from controls (Student's t-test p < 0.005). [file 1471-213X-8-120-S2.jpeg]
